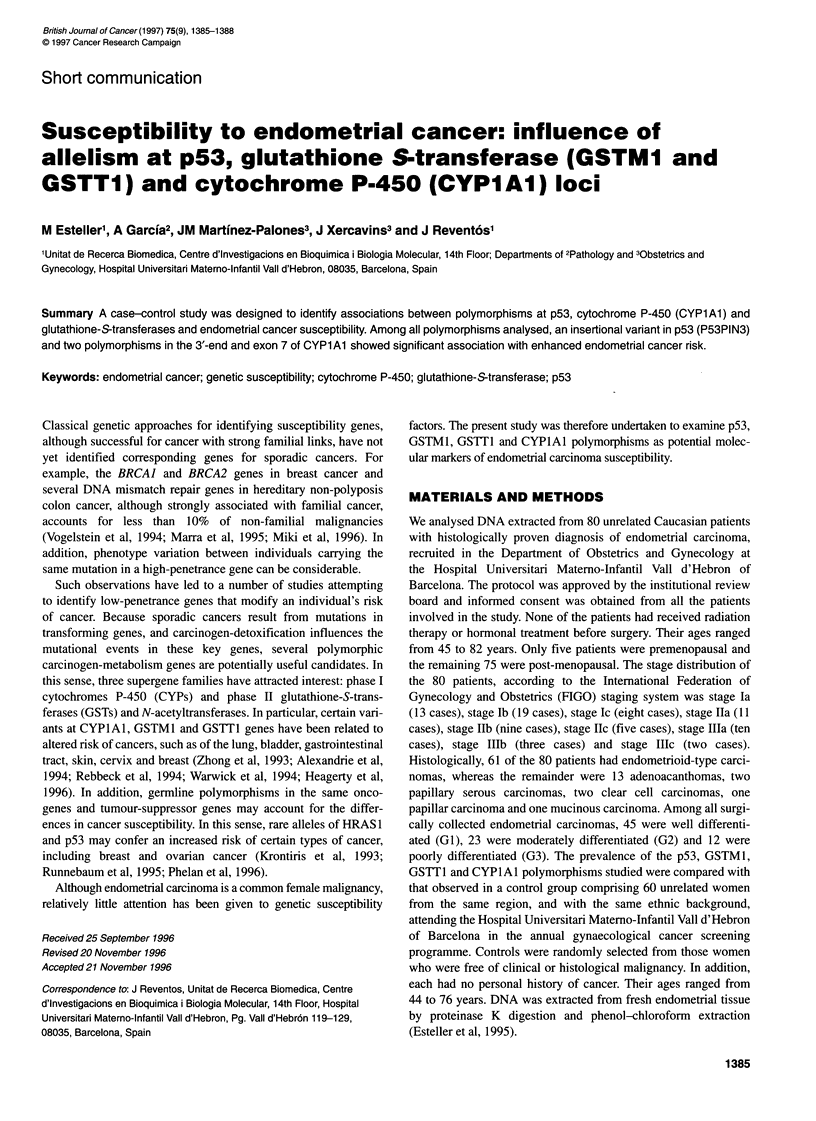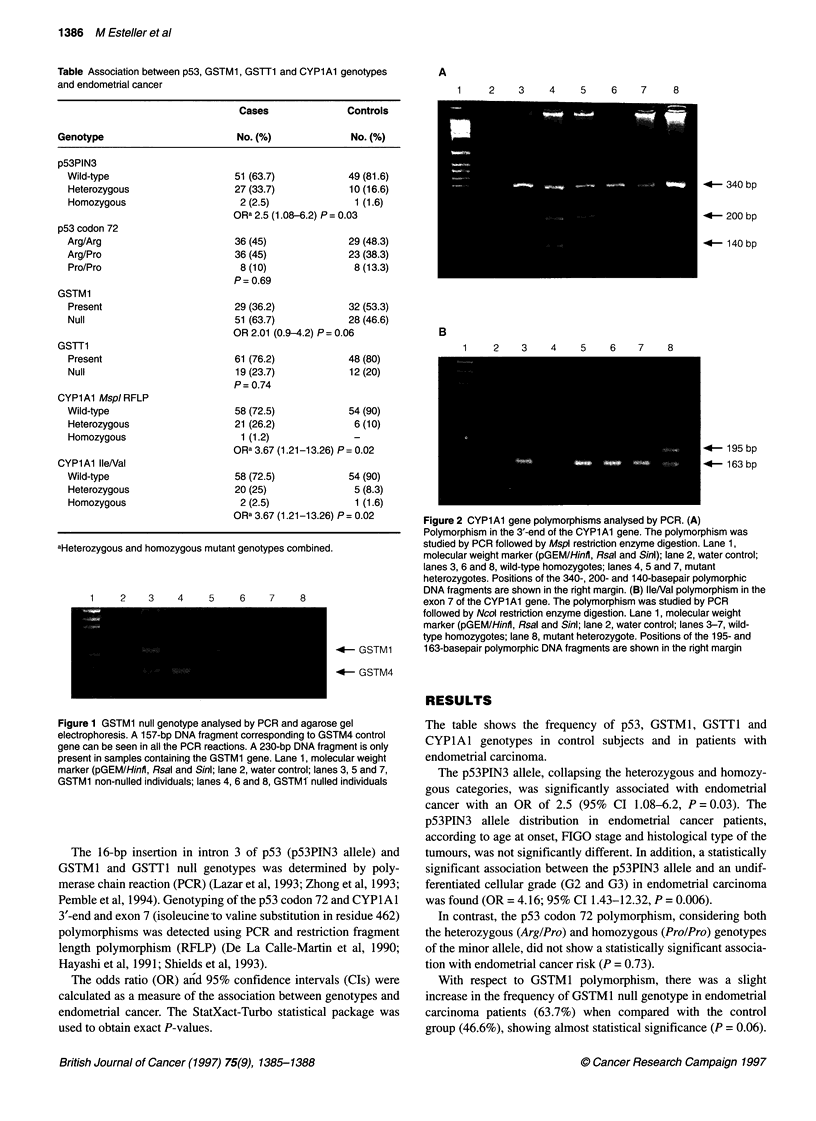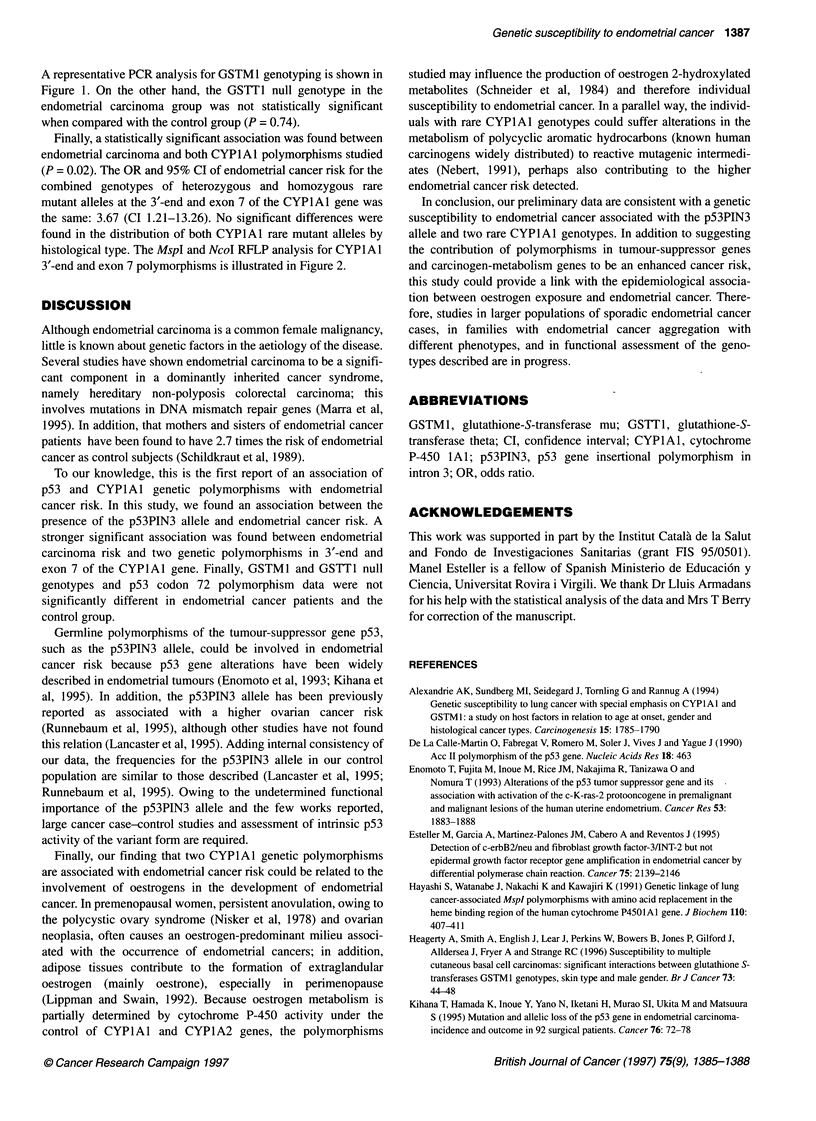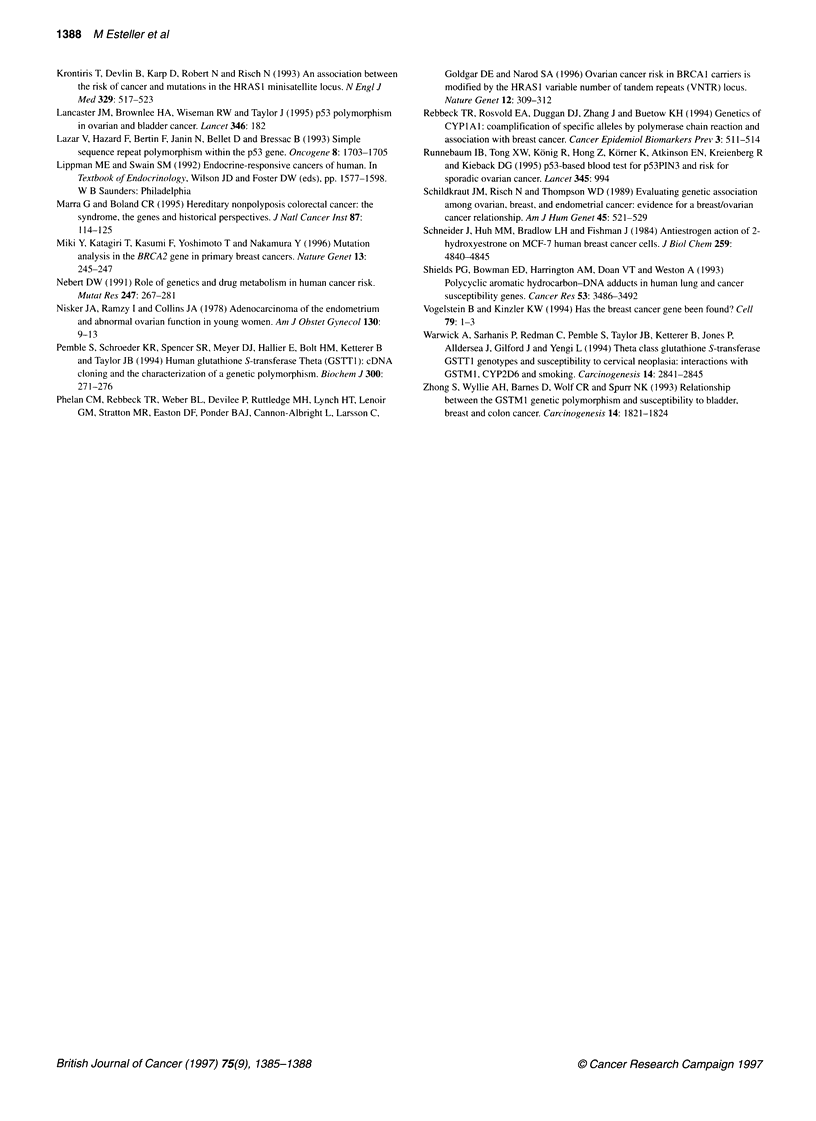# Susceptibility to endometrial cancer: influence of allelism at p53, glutathione S-transferase (GSTM1 and GSTT1) and cytochrome P-450 (CYP1A1) loci.

**DOI:** 10.1038/bjc.1997.235

**Published:** 1997

**Authors:** M. Esteller, A. GarcÃ­a, J. M. MartÃ­nez-Palones, J. Xercavins, J. ReventÃ³s

**Affiliations:** Unitat de Recerca Biomedica, Centre d'Investigacions en Bioquimica i Biologia Molecular, Hospital Universitari Materno-Infantil Vall d'Hebron, Barcelona, Spain.

## Abstract

**Images:**


British Journal of Cancer (1997) 75(9), 1385–1388
© 1997 Cancer Research Campaign

Short communication

### Susceptibility to endometrial cancer: influence of allelism at p53, glutathione *S*-transferase (GSTM1 and GSTT1) and cytochrome P-450 (CYP1A1) loci

**M Esteller[1], A García[2], JM Martínez-Palones[3], J Xercavins[3] and J Reventós[1]**

[1]Unitat de Recerca Biomedica, Centre d'Investigacions en Bioquimica i Biologia Molecular, 14th Floor; Departments of [2]Pathology and [3]Obstetrics and Gynecology, Hospital Universitari Materno-Infantil Vall d'Hebron, 08035, Barcelona, Spain

**Summary** A case–control study was designed to identify associations between polymorphisms at p53, cytochrome P-450 (CYP1A1) and glutathione-*S*-transferases and endometrial cancer susceptibility. Among all polymorphisms analysed, an insertional variant in p53 (P53PIN3) and two polymorphisms in the 3′-end and exon 7 of CYP1A1 showed significant association with enhanced endometrial cancer risk.

**Keywords:** endometrial cancer; genetic susceptibility; cytochrome P-450; glutathione-*S*-transferase; p53

Classical genetic approaches for identifying susceptibility genes, although successful for cancer with strong familial links, have not yet identified corresponding genes for sporadic cancers. For example, the *BRCA1* and *BRCA2* genes in breast cancer and several DNA mismatch repair genes in hereditary non-polyposis colon cancer, although strongly associated with familial cancer, accounts for less than 10% of non-familial malignancies (Vogelstein et al, 1994; Marra et al, 1995; Miki et al, 1996). In addition, phenotype variation between individuals carrying the same mutation in a high-penetrance gene can be considerable.

Such observations have led to a number of studies attempting to identify low-penetrance genes that modify an individual's risk of cancer. Because sporadic cancers result from mutations in transforming genes, and carcinogen-detoxification influences the mutational events in these key genes, several polymorphic carcinogen-metabolism genes are potentially useful candidates. In this sense, three supergene families have attracted interest: phase I cytochromes P-450 (CYPs) and phase II glutathione-*S*-transferases (GSTs) and *N*-acetyltransferases. In particular, certain variants at CYP1A1, GSTM1 and GSTT1 genes have been related to altered risk of cancers, such as of the lung, bladder, gastrointestinal tract, skin, cervix and breast (Zhong et al, 1993; Alexandrie et al, 1994; Rebbeck et al, 1994; Warwick et al, 1994; Heagerty et al, 1996). In addition, germline polymorphisms in the same oncogenes and tumour-suppressor genes may account for the differences in cancer susceptibility. In this sense, rare alleles of HRAS1 and p53 may confer an increased risk of certain types of cancer, including breast and ovarian cancer (Krontiris et al, 1993; Runnebaum et al, 1995; Phelan et al, 1996).

Although endometrial carcinoma is a common female malignancy, relatively little attention has been given to genetic susceptibility factors. The present study was therefore undertaken to examine p53, GSTM1, GSTT1 and CYP1A1 polymorphisms as potential molecular markers of endometrial carcinoma susceptibility.

*Received 25 September 1996*
*Revised 20 November 1996*
*Accepted 21 November 1996*

*Correspondence to*: J Reventos, Unitat de Recerca Biomedica, Centre d'Investigacions en Bioquimica i Biologia Molecular, 14th Floor, Hospital Universitari Materno-Infantil Vall d'Hebron, Pg. Vall d'Hebrón 119–129, 08035, Barcelona, Spain

### MATERIALS AND METHODS

We analysed DNA extracted from 80 unrelated Caucasian patients with histologically proven diagnosis of endometrial carcinoma, recruited in the Department of Obstetrics and Gynecology at the Hospital Universitari Materno-Infantil Vall d'Hebron of Barcelona. The protocol was approved by the institutional review board and informed consent was obtained from all the patients involved in the study. None of the patients had received radiation therapy or hormonal treatment before surgery. Their ages ranged from 45 to 82 years. Only five patients were premenopausal and the remaining 75 were post-menopausal. The stage distribution of the 80 patients, according to the International Federation of Gynecology and Obstetrics (FIGO) staging system was stage Ia (13 cases), stage Ib (19 cases), stage Ic (eight cases), stage IIa (11 cases), stage IIb (nine cases), stage IIc (five cases), stage IIIa (ten cases), stage IIIb (three cases) and stage IIIc (two cases). Histologically, 61 of the 80 patients had endometrioid-type carcinomas, whereas the remainder were 13 adenoacanthomas, two papillary serous carcinomas, two clear cell carcinomas, one papillar carcinoma and one mucinous carcinoma. Among all surgically collected endometrial carcinomas, 45 were well differentiated (G1), 23 were moderately differentiated (G2) and 12 were poorly differentiated (G3). The prevalence of the p53, GSTM1, GSTT1 and CYP1A1 polymorphisms studied were compared with that observed in a control group comprising 60 unrelated women from the same region, and with the same ethnic background, attending the Hospital Universitari Materno-Infantil Vall d'Hebron of Barcelona in the annual gynaecological cancer screening programme. Controls were randomly selected from those women who were free of clinical or histological malignancy. In addition, each had no personal history of cancer. Their ages ranged from 44 to 76 years. DNA was extracted from fresh endometrial tissue by proteinase K digestion and phenol–chloroform extraction (Esteller et al, 1995).

**Table** Association between p53, GSTM1, GSTT1 and CYP1A1 genotypes and endometrial cancer

| Genotype | Cases No. (%) | Controls No. (%) |
|---|---|---|
| p53PIN3 | | |
| Wild-type | 51 (63.7) | 49 (81.6) |
| Heterozygous | 27 (33.7) | 10 (16.6) |
| Homozygous | 2 (2.5) | 1 (1.6) |
| | OR[a] 2.5 (1.08–6.2) *P* = 0.03 | |
| p53 codon 72 | | |
| Arg/Arg | 36 (45) | 29 (48.3) |
| Arg/Pro | 36 (45) | 23 (38.3) |
| Pro/Pro | 8 (10) | 8 (13.3) |
| | *P* = 0.69 | |
| GSTM1 | | |
| Present | 29 (36.2) | 32 (53.3) |
| Null | 51 (63.7) | 28 (46.6) |
| | OR 2.01 (0.9–4.2) *P* = 0.06 | |
| GSTT1 | | |
| Present | 61 (76.2) | 48 (80) |
| Null | 19 (23.7) | 12 (20) |
| | *P* = 0.74 | |
| CYP1A1 *MspI* RFLP | | |
| Wild-type | 58 (72.5) | 54 (90) |
| Heterozygous | 21 (26.2) | 6 (10) |
| Homozygous | 1 (1.2) | – |
| | OR[a] 3.67 (1.21–13.26) *P* = 0.02 | |
| CYP1A1 Ile/Val | | |
| Wild-type | 58 (72.5) | 54 (90) |
| Heterozygous | 20 (25) | 5 (8.3) |
| Homozygous | 2 (2.5) | 1 (1.6) |
| | OR[a] 3.67 (1.21–13.26) *P* = 0.02 | |

[a]Heterozygous and homozygous mutant genotypes combined.

**Figure 1** GSTM1 null genotype analysed by PCR and agarose gel electrophoresis. A 157-bp DNA fragment corresponding to GSTM4 control gene can be seen in all the PCR reactions. A 230-bp DNA fragment is only present in samples containing the GSTM1 gene. Lane 1, molecular weight marker (pGEM/*Hinf*I, *Rsa*I and *Sin*I; lane 2, water control; lanes 3, 5 and 7, GSTM1 non-nulled individuals; lanes 4, 6 and 8, GSTM1 nulled individuals

The 16-bp insertion in intron 3 of p53 (p53PIN3 allele) and GSTM1 and GSTT1 null genotypes was determined by polymerase chain reaction (PCR) (Lazar et al, 1993; Zhong et al, 1993; Pemble et al, 1994). Genotyping of the p53 codon 72 and CYP1A1 3′-end and exon 7 (isoleucine to valine substitution in residue 462) polymorphisms was detected using PCR and restriction fragment length polymorphism (RFLP) (De La Calle-Martin et al, 1990; Hayashi et al, 1991; Shields et al, 1993).

The odds ratio (OR) and 95% confidence intervals (CIs) were calculated as a measure of the association between genotypes and endometrial cancer. The StatXact-Turbo statistical package was used to obtain exact *P*-values.

**Figure 2** CYP1A1 gene polymorphisms analysed by PCR. (**A**) Polymorphism in the 3′-end of the CYP1A1 gene. The polymorphism was studied by PCR followed by *Msp*I restriction enzyme digestion. Lane 1, molecular weight marker (pGEM/*Hinf*I, *Rsa*I and *Sin*I); lane 2, water control; lanes 3, 6 and 8, wild-type homozygotes; lanes 4, 5 and 7, mutant heterozygotes. Positions of the 340-, 200- and 140-basepair polymorphic DNA fragments are shown in the right margin. (**B**) Ile/Val polymorphism in the exon 7 of the CYP1A1 gene. The polymorphism was studied by PCR followed by *Nco*I restriction enzyme digestion. Lane 1, molecular weight marker (pGEM/*Hinf*I, *Rsa*I and *Sin*I; lane 2, water control; lanes 3–7, wild-type homozygotes; lane 8, mutant heterozygote. Positions of the 195- and 163-basepair polymorphic DNA fragments are shown in the right margin

### RESULTS

The table shows the frequency of p53, GSTM1, GSTT1 and CYP1A1 genotypes in control subjects and in patients with endometrial carcinoma.

The p53PIN3 allele, collapsing the heterozygous and homozygous categories, was significantly associated with endometrial cancer with an OR of 2.5 (95% CI 1.08–6.2, *P* = 0.03). The p53PIN3 allele distribution in endometrial cancer patients, according to age at onset, FIGO stage and histological type of the tumours, was not significantly different. In addition, a statistically significant association between the p53PIN3 allele and an undifferentiated cellular grade (G2 and G3) in endometrial carcinoma was found (OR = 4.16; 95% CI 1.43–12.32, *P* = 0.006).

In contrast, the p53 codon 72 polymorphism, considering both the heterozygous (*Arg/Pro*) and homozygous (*Pro/Pro*) genotypes of the minor allele, did not show a statistically significant association with endometrial cancer risk (*P* = 0.73).

With respect to GSTM1 polymorphism, there was a slight increase in the frequency of GSTM1 null genotype in endometrial carcinoma patients (63.7%) when compared with the control group (46.6%), showing almost statistical significance (*P* = 0.06).

 *© Cancer Research Campaign 1997*

A representative PCR analysis for GSTM1 genotyping is shown in Figure 1. On the other hand, the GSTT1 null genotype in the endometrial carcinoma group was not statistically significant when compared with the control group ($P = 0.74$).

Finally, a statistically significant association was found between endometrial carcinoma and both CYP1A1 polymorphisms studied ($P = 0.02$). The OR and 95% CI of endometrial cancer risk for the combined genotypes of heterozygous and homozygous rare mutant alleles at the 3′-end and exon 7 of the CYP1A1 gene was the same: 3.67 (CI 1.21–13.26). No significant differences were found in the distribution of both CYP1A1 rare mutant alleles by histological type. The *Msp*I and *Nco*I RFLP analysis for CYP1A1 3′-end and exon 7 polymorphisms is illustrated in Figure 2.

### DISCUSSION

Although endometrial carcinoma is a common female malignancy, little is known about genetic factors in the aetiology of the disease. Several studies have shown endometrial carcinoma to be a significant component in a dominantly inherited cancer syndrome, namely hereditary non-polyposis colorectal carcinoma; this involves mutations in DNA mismatch repair genes (Marra et al, 1995). In addition, that mothers and sisters of endometrial cancer patients have been found to have 2.7 times the risk of endometrial cancer as control subjects (Schildkraut et al, 1989).

To our knowledge, this is the first report of an association of p53 and CYP1A1 genetic polymorphisms with endometrial cancer risk. In this study, we found an association between the presence of the p53PIN3 allele and endometrial cancer risk. A stronger significant association was found between endometrial carcinoma risk and two genetic polymorphisms in 3′-end and exon 7 of the CYP1A1 gene. Finally, GSTM1 and GSTT1 null genotypes and p53 codon 72 polymorphism data were not significantly different in endometrial cancer patients and the control group.

Germline polymorphisms of the tumour-suppressor gene p53, such as the p53PIN3 allele, could be involved in endometrial cancer risk because p53 gene alterations have been widely described in endometrial tumours (Enomoto et al, 1993; Kihana et al, 1995). In addition, the p53PIN3 allele has been previously reported as associated with a higher ovarian cancer risk (Runnebaum et al, 1995), although other studies have not found this relation (Lancaster et al, 1995). Adding internal consistency of our data, the frequencies for the p53PIN3 allele in our control population are similar to those described (Lancaster et al, 1995; Runnebaum et al, 1995). Owing to the undetermined functional importance of the p53PIN3 allele and the few works reported, large cancer case–control studies and assessment of intrinsic p53 activity of the variant form are required.

Finally, our finding that two CYP1A1 genetic polymorphisms are associated with endometrial cancer risk could be related to the involvement of oestrogens in the development of endometrial cancer. In premenopausal women, persistent anovulation, owing to the polycystic ovary syndrome (Nisker et al, 1978) and ovarian neoplasia, often causes an oestrogen-predominant milieu associated with the occurrence of endometrial cancers; in addition, adipose tissues contribute to the formation of extraglandular oestrogen (mainly oestrone), especially in perimenopause (Lippman and Swain, 1992). Because oestrogen metabolism is partially determined by cytochrome P-450 activity under the control of CYP1A1 and CYP1A2 genes, the polymorphisms studied may influence the production of oestrogen 2-hydroxylated metabolites (Schneider et al, 1984) and therefore individual susceptibility to endometrial cancer. In a parallel way, the individuals with rare CYP1A1 genotypes could suffer alterations in the metabolism of polycyclic aromatic hydrocarbons (known human carcinogens widely distributed) to reactive mutagenic intermediates (Nebert, 1991), perhaps also contributing to the higher endometrial cancer risk detected.

In conclusion, our preliminary data are consistent with a genetic susceptibility to endometrial cancer associated with the p53PIN3 allele and two rare CYP1A1 genotypes. In addition to suggesting the contribution of polymorphisms in tumour-suppressor genes and carcinogen-metabolism genes to be an enhanced cancer risk, this study could provide a link with the epidemiological association between oestrogen exposure and endometrial cancer. Therefore, studies in larger populations of sporadic endometrial cancer cases, in families with endometrial cancer aggregation with different phenotypes, and in functional assessment of the genotypes described are in progress.

### ABBREVIATIONS

GSTM1, glutathione-*S*-transferase mu; GSTT1, glutathione-*S*-transferase theta; CI, confidence interval; CYP1A1, cytochrome P-450 1A1; p53PIN3, p53 gene insertional polymorphism in intron 3; OR, odds ratio.

### ACKNOWLEDGEMENTS

This work was supported in part by the Institut Català de la Salut and Fondo de Investigaciones Sanitarias (grant FIS 95/0501). Manel Esteller is a fellow of Spanish Ministerio de Educación y Ciencia, Universitat Rovira i Virgili. We thank Dr Lluis Armadans for his help with the statistical analysis of the data and Mrs T Berry for correction of the manuscript.

### REFERENCES

Alexandrie AK, Sundberg MI, Seidegard J, Tornling G and Rannug A (1994) Genetic susceptibility to lung cancer with special emphasis on CYP1A1 and GSTM1: a study on host factors in relation to age at onset, gender and histological cancer types. *Carcinogenesis* **15**: 1785–1790

De La Calle-Martin O, Fabregat V, Romero M, Soler J, Vives J and Yague J (1990) Acc II polymorphism of the p53 gene. *Nucleic Acids Res* **18**: 463

Enomoto T, Fujita M, Inoue M, Rice JM, Nakajima R, Tanizawa O and Nomura T (1993) Alterations of the p53 tumor suppressor gene and its association with activation of the c-K-ras-2 protooncogene in premalignant and malignant lesions of the human uterine endometrium. *Cancer Res* **53**: 1883–1888

Esteller M, Garcia A, Martinez-Palones JM, Cabero A and Reventos J (1995) Detection of c-erbB2/neu and fibroblast growth factor-3/INT-2 but not epidermal growth factor receptor gene amplification in endometrial cancer by differential polymerase chain reaction. *Cancer* **75**: 2139–2146

Hayashi S, Watanabe J, Nakachi K and Kawajiri K (1991) Genetic linkage of lung cancer-associated *MspI* polymorphisms with amino acid replacement in the heme binding region of the human cytochrome P4501A1 gene. *J Biochem* **110**: 407–411

Heagerty A, Smith A, English J, Lear J, Perkins W, Bowers B, Jones P, Gilford J, Alldersea J, Fryer A and Strange RC (1996) Susceptibility to multiple cutaneous basal cell carcinomas: significant interactions between glutathione *S*-transferases GSTM1 genotypes, skin type and male gender. *Br J Cancer* **73**: 44–48

Kihana T, Hamada K, Inoue Y, Yano N, Iketani H, Murao SI, Ukita M and Matsuura S (1995) Mutation and allelic loss of the p53 gene in endometrial carcinoma-incidence and outcome in 92 surgical patients. *Cancer* **76**: 72–78

© Cancer Research Campaign 1997

Krontiris T, Devlin B, Karp D, Robert N and Risch N (1993) An association between the risk of cancer and mutations in the HRAS1 minisatellite locus. *N Engl J Med* **329**: 517–523

Lancaster JM, Brownlee HA, Wiseman RW and Taylor J (1995) p53 polymorphism in ovarian and bladder cancer. *Lancet* **346**: 182

Lazar V, Hazard F, Bertin F, Janin N, Bellet D and Bressac B (1993) Simple sequence repeat polymorphism within the p53 gene. *Oncogene* **8**: 1703–1705

Lippman ME and Swain SM (1992) Endocrine-responsive cancers of human. In *Textbook of Endocrinology*, Wilson JD and Foster DW (eds), pp. 1577–1598. W B Saunders: Philadelphia

Marra G and Boland CR (1995) Hereditary nonpolyposis colorectal cancer: the syndrome, the genes and historical perspectives. *J Natl Cancer Inst* **87**: 114–125

Miki Y, Katagiri T, Kasumi F, Yoshimoto T and Nakamura Y (1996) Mutation analysis in the *BRCA2* gene in primary breast cancers. *Nature Genet* **13**: 245–247

Nebert DW (1991) Role of genetics and drug metabolism in human cancer risk. *Mutat Res* **247**: 267–281

Nisker JA, Ramzy I and Collins JA (1978) Adenocarcinoma of the endometrium and abnormal ovarian function in young women. *Am J Obstet Gynecol* **130**: 9–13

Pemble S, Schroeder KR, Spencer SR, Meyer DJ, Hallier E, Bolt HM, Ketterer B and Taylor JB (1994) Human glutathione *S*-transferase Theta (GSTT1): cDNA cloning and the characterization of a genetic polymorphism. *Biochem J* **300**: 271–276

Phelan CM, Rebbeck TR, Weber BL, Devilee P, Ruttledge MH, Lynch HT, Lenoir GM, Stratton MR, Easton DF, Ponder BAJ, Cannon-Albright L, Larsson C, Goldgar DE and Narod SA (1996) Ovarian cancer risk in BRCA1 carriers is modified by the HRAS1 variable number of tandem repeats (VNTR) locus. *Nature Genet* **12**: 309–312

Rebbeck TR, Rosvold EA, Duggan DJ, Zhang J and Buetow KH (1994) Genetics of CYP1A1: coamplification of specific alleles by polymerase chain reaction and association with breast cancer. *Cancer Epidemiol Biomarkers Prev* **3**: 511–514

Runnebaum IB, Tong XW, König R, Hong Z, Körner K, Atkinson EN, Kreienberg R and Kieback DG (1995) p53-based blood test for p53PIN3 and risk for sporadic ovarian cancer. *Lancet* **345**: 994

Schildkraut JM, Risch N and Thompson WD (1989) Evaluating genetic association among ovarian, breast, and endometrial cancer: evidence for a breast/ovarian cancer relationship. *Am J Hum Genet* **45**: 521–529

Schneider J, Huh MM, Bradlow LH and Fishman J (1984) Antiestrogen action of 2-hydroxyestrone on MCF-7 human breast cancer cells. *J Biol Chem* **259**: 4840–4845

Shields PG, Bowman ED, Harrington AM, Doan VT and Weston A (1993) Polycyclic aromatic hydrocarbon–DNA adducts in human lung and cancer susceptibility genes. *Cancer Res* **53**: 3486–3492

Vogelstein B and Kinzler KW (1994) Has the breast cancer gene been found? *Cell* **79**: 1–3

Warwick A, Sarhanis P, Redman C, Pemble S, Taylor JB, Ketterer B, Jones P, Alldersea J, Gilford J and Yengi L (1994) Theta class glutathione *S*-transferase GSTT1 genotypes and susceptibility to cervical neoplasia: interactions with GSTM1, CYP2D6 and smoking. *Carcinogenesis* **14**: 2841–2845

Zhong S, Wyllie AH, Barnes D, Wolf CR and Spurr NK (1993) Relationship between the GSTM1 genetic polymorphism and susceptibility to bladder, breast and colon cancer. *Carcinogenesis* **14**: 1821–1824

*© Cancer Research Campaign 1997*